# Exploring the Prevalence and Coexistence of Metabolic Dysfunction-associated Steatotic Liver Disease in Type 2 Diabetes Mellitus Patients Using Ultrasound: A Cross-sectional Study

**DOI:** 10.2174/0115734056354807241217043210

**Published:** 2025-01-02

**Authors:** Ibrahim Hadadi, Mohamed Adam, Mustafa J Musa, Awadia Gareeballah, Mansour Alqahtani, Ibrahem Kanbayti, Ahmed Hazazi

**Affiliations:** 1 Department of Radiological Sciences, College of Applied Medical Sciences, King Khalid University, Abha, Asir, Saudi Arabia; 2 Department of Applied Radiologic Science, University of Jeddah, Jeddah, Saudi Arabia; 3 Department of Diagnostic Radiology Technology, College of Applied Medical Sciences, Taibah University, Al-Madinah Al-Munawwarrah, Saudi Arabia; 4 Department of Radiological Sciences, College of Applied Medical Sciences, Najran University, Najran, Saudi Arabia; 5 Radiologic Sciences Department, Faculty of Applied Medical Sciences, King Abdulaziz University, Jeddah, Saudi Arabia; 6 Department of Public Health, Faculty of Health Science, Saudi Electronic University, Riyadh, Saudi Arabia

**Keywords:** Ultrasound, Type 2 diabetic mellitus, MASLD, Uncontrolled diabetes, Controlled diabetes, BMI

## Abstract

**Background:**

Type 2 diabetes Mellitus (T2DM) increases vulnerability to metabolic dysfunction-associated steatotic liver disease (MASLD). Therefore, this study aims to determine the prevalence and coexistence of MASLD in patients with T2DM using ultrasound.

**Methods:**

This cross-sectional retrospective study included 168 patients with T2DM from multiple diabetes clinics in Abha City, Asir region, recruited between August 2023 and December 2023. Adult patients aged 18 and over with T2DM were included, and data was extracted from patient files. All patients were examined by ultrasound to determine the prevalence and coexistence of MASLD in patients with T2DM. Hepatic steatosis on B-mode ultrasound is qualitatively classified on a four-point scale: normal (0), mild (1), moderate (2), and severe (3).

**Results:**

Out of 168 patients, 68.4% were identified with MASLD, mostly with diffuse liver (97.4%) diagnosed through ultrasound. MASLD was significantly higher in individuals with uncontrolled diabetes (72.5%) than those with controlled diabetes (46.2%), with a significant difference (*p*=0.015) and an odds ratio (OR) of 3.081, indicating uncontrolled diabetics are over three times more likely to develop MASLD. The uncontrolled group had a statistically significant larger liver size than the control group (13.6cm ±1.43 *vs*. 13.0cm ±1.20, respectively: [*p*=0.032, 95%CI 0.053-1.12]). Furthermore, a notable association was observed between increased BMI and the prevalence of MASLD in individuals with T2DM. Furthermore, no significant association was found between the duration of diabetes and the severity of MASLD, nor between the grading of MASLD and gender.

**Conclusion:**

This study highlights a crucial association between uncontrolled diabetes and increased MASLD prevalence, emphasizing the importance of diabetes management in reducing MASLD risk.

## 1 INTRODUCTION

Metabolic dysfunction-associated steatotic liver disease (MASLD), previously referred to as nonalcoholic fatty liver disease (NAFLD), is a common chronic liver condition [1]. MASLD arises from complex interactions involving multiple cardiometabolic and environmental risk factors, making it a multifactorial disease [2]. The global prevalence of MASLD among patients with Type 2 Diabetes Mellitus (T2DM) is approximately 55.5%, with Europe and West Asia reporting the highest rates at 68.0% and 67.3%, respectively, and Africa showing the lowest prevalence at 30.4% [3]. MASLD is defined by excessive fat deposition in the liver, commonly linked with insulin resistance, and characterized by a liver fat content exceeding 5% in hepatocytes. Frequently, MASLD is correlated with the occurrence of metabolic syndrome and its various components, factors that elevate the risk of progression to more advanced and severe stages of the disease [4].

The coexistence of MASLD and T2DM is a common clinical phenomenon [5]. In patients with T2DM, the prevalence of MASLD stands at 59.67%. This comorbidity is associated with unfavorable outcomes, including an increased mortality rate attributed to cirrhosis [6]. Among individuals diagnosed with T2DM, this prevalence escalates to 55–80% [7, 8]. Given these high prevalence rates, contemporary guidelines advocate for routine screening of all T2DM patients for MASLD, focusing on detecting advanced fibrosis [9, 10].

Histological examination significantly confirms the extent of fibrosis staging, the degree of steatosis, and the scope of inflammation. It also plays a crucial role in excluding autoimmune hepatitis. While liver biopsy is a clinically recognized and generally safe method, its invasive nature carries potential risks, including a mortality rate of up to 0.2%. Post-procedure pain, reported in up to 84% of cases, often necessitates inpatient hospitalization, leading to relatively low patient acceptance of this procedure [11, 12].

Per the latest guidelines from the European Association for the Study of the Liver (EASL), Magnetic Resonance Imaging (MRI) utilizing the proton density fat fraction (PDFF) is now regarded as comparable to biopsy for assessing liver fat content (“EASL-EASD-EASO Clinical Practice Guidelines for the management of non-alcoholic fatty liver disease,” 2016). However, limitations of MRI include its limited availability, substantial costs, and considerable time requirement. Ultrasound represents another non-invasive imaging modality, favored as a first-line diagnostic tool indicating increased echogenicity in MASLD [13]. Hernaez *et al*. (2011) conducted a meta-analysis examining how accurately ultrasound detects MASLD. The analysis revealed that ultrasound achieves a sensitivity of around 84.8% and a specificity of about 93.6%, highlighting its reliability as a non-invasive method for diagnosing MASLD [14]. Additionally, ultrasound is cost-effective, broadly accessible, without side effects, and can be performed repeatedly. It also enjoys high patient acceptance, partly due to the direct engagement with the operator during the examination [15, 16].

In routine outpatient practice, the concurrent presence of MASLD and T2DM is frequently observed. While MASLD alone is associated with a relatively low risk of severe complications, its co-occurrence with T2DM markedly exacerbates the patient's prognosis. It is essential to screen for T2DM in individuals diagnosed with MASLD. Conversely, current clinical guidelines do not mandate routine screening for MASLD in patients already diagnosed with diabetes [17].

Recent studies highlight the high prevalence of MASLD in T2DM populations and the significant risk it poses for metabolic and cardiovascular complications. A review by Ciardullo *et al*. (2023) reported a strong association between T2DM and advanced stages of MASLD, underscoring the urgent need for effective screening methods within diabetic populations [18]. Building on this existing body of literature, our study focuses specifically on the Saudi population, providing insight into regional variations in MASLD prevalence among T2DM patients.

This study emphasizes the importance of incorporating MASLD screening into routine clinical care for diabetic patients in Saudi Arabia, using ultrasound as a practical and accessible diagnostic tool. By examining the prevalence of MASLD in T2DM cohorts, our research highlights the value of timely detection, prevention, and management of hepatic impairment, aiming to improve patient outcomes and reduce the burden of liver-related complications in diabetic populations.

## 2 MATERIALS AND METHODS

### 2.1 Ethical Approval

The study received ethical approval from the Research Ethics Committee, College of Medicine, King Khalid University (HA-O7-B-012). Informed consent was obtained from all the recruited patients, Saudi Arabia.

### 2.2 Study Design and Data Collection

This cross-sectional retrospective study included 168 patients with T2DM from multiple di-abetes clinics (seven clinics) in Abha City, Asir region, recruited between August 2023 and December 2023. Patients were selected using a systematic random sampling method, ensuring a high degree of randomization across the sample. Data collection encompassed clinical and sociodemographic details, including age, gender, duration of diabetes, pharmacological and medical history, and diabetes treatment modalities extracted from patient records. Body Mass Index (BMI) was calculated using the formula weight (kg) divided by height (m^2^). BMI categories were defined as normal (18.5 to 24.9), overweight (25 to 29.9), obesity class I (30 to 34.9), obesity class II (35-39.9) and obesity class III (40 and above).

### 2.3 Inclusion and Exclusion Criteria

The primary inclusion criterion was adult patients aged 18 years or older diagnosed with T2DM. Exclusion criteria for this study encompassed patients with pre-existing hepatobiliary disorders, excluding MASLD, as well as those with malignancies, ascites, or a history of inflammatory bowel disease. Furthermore, individuals prescribed pharmaceuticals known to induce hepatic steatosis, like estrogens, corticosteroids, amiodarone, or valproate, either presently or in the previous 12 months, were likewise ineligible for inclusion.

### 2.4 Ultrasound Examination

The examinations followed a standardized protocol where patients were positioned supine, utilizing a high-resolution ultrasound machine (Mindray DC-70) equipped with a 3.5 MHz convex transducer. Prior to the procedure, patients were instructed to fast for a minimum of 6 hours to ensure optimal imaging quality. All ultrasound examinations were referred to a single, experienced radiologist by the participating clinics, ensuring consistent documentation of the liver's texture and dimensions across all cases.

The assessment of hepatic steatosis *via* conventional B-mode ultrasound relies on observing alterations in the echo pattern of liver parenchyma. This change occurs as fat vacuoles within hepatocytes reflect the ultrasound beam, leading to an increase in hepatic parenchymal echogenicity that corresponds with the progression of hepatic steatosis. Consequently, the liver exhibits a “bright liver” appearance in B-mode US imaging.

For evaluating hepatic parenchymal echogenicity, the echogenicity of the right kidney cortex serves as an internal reference. Hepatic steatosis is suspected when the liver parenchymal echogenicity surpasses that of the right kidney cortex. The extent of hepatic steatosis observed in B-mode US is typically classified qualitatively into a four-point scale: normal (grade 0), mild (grade 1), moderate (grade 2), and severe (grade 3) [19], as shown in Fig. (**1**).

#### 2.4.1 Grade 0

Liver echogenicity is similar to that of the renal cortex.

#### 2.4.2 Grade 1

The liver appears slightly brighter than the renal cortex, with the diaphragm and hepatic vein interfaces remaining clearly visible and sharply contoured.

#### 2.4.3 Grade 2

Enhanced liver brightness with attenuated ultrasound beam in the deeper liver sections; the diaphragm and hepatic veins are visible but exhibit blunted contours.

#### 2.4.4 Grade 3

The liver presents with a bright appearance, significant ultrasound beam attenuation, and obscured visibility of the diaphragm and hepatic veins.

### 2.5 Statistical Analysis

The statistical analysis was performed using SPSS version 27 for Windows (SPSS Inc., Chicago, IL, USA). Descriptive statistics determined the mean ± standard deviation for numerical values. The normality of the data distribution was examined using the Shapiro-Wilk test to confirm the suitability of parametric statistical tests. Crosstabulation analysis was conducted to explore the relationship between MASLD and patients categorized as having controlled *versus* uncontrolled Diabetes Mellitus (DM), and the odds ratio (OR) was computed to offer insights into the likelihood of MASLD occurrence. The research delved into the association between variables (duration of DM, gender, and BMI) and MASLD grading (severity). An independent sample t-test was employed to assess potential differences in liver size between patients with uncontrolled and controlled DM. Statistical significance was established with a *p*-value of ≤ 0.05.

## 3 RESULTS

The study included 168 T2DM patients, 52.4% male and 47.6% female, with a mean age of 57.3 years and a BMI of 30.2. Most patients were 40-59 years old, accounting for 43.5%, and only 6.5% aged 80-99. Only 6.6% of patients lie in obesity class III; among the others, 24.4% were normal, 23.8% were categorized as overweight, while 25.6% lie in obesity class I and 19.6% in obesity class III. Most patients have primary education, accounting for 61.9%, and only 4.2% were illiterate. Most were non-smokers, accounting for 78%, while only 7.1% were ex-smokers. Oral hypoglycemia was the most common treatment, accounting for 46.4%, while 1.8% had no treatment. Most have diabetes for 1-10 years, accounting for 48.2%, while 10.1% have more than 20 years. Uncontrolled diabetes status was observed in 84.5% of patients, and controlled were 15.5%. Most patients were married, accounting for 79.2%, 7.1% were single, 1.2% were divorced, and 12.5% were widows, as shown in Table **1**.

Out of the 168 patients, 115 (68.4%) were diagnosed with fatty liver using ultrasound. Among these, 97.4% (112 patients) had diffuse fatty liver, whereas 2.6% (3 patients) had focal fatty liver. Among MASLD patients, 80 out of 115 had G I fatty liver (69.6%), 32 had G II fatty liver (27.8%), and 3 patients had G III fatty liver (2.6%), as shown in Table **2**.

The data presented in Table **3**. shows that 103 out of 142 patients with uncontrolled diabetes had fatty liver. In contrast, only 12 out of 26 patients with controlled diabetes had fatty liver. This significant difference (*p*-value = 0.015) suggesting a higher prevalence of fatty liver in individuals with uncontrolled diabetes. The OR of 3.081 further emphasizes this disparity, revealing that individuals with uncontrolled diabetes were more than three times as likely to have fatty liver compared to those with controlled diabetes.

The study found no significant association between the duration of diabetes mellitus and the grading of MASLD (*p* = 0.297), as shown in Table **4**. Moreover, the liver size was larger in uncontrolled DM than in the control group. There was a significant difference in the average liver length between the two groups (13.6 cm ± 1.43 in the uncontrolled DM group *versus* 13.00 cm ± 1.2 in the control group; *p* = 0.032, 95% CI 0.053-1.12) as shown in Table **5** and Fig. (**2**).

There was no significant association between the MASLD grading and gender (*p* = 0.525), as shown in Fig. (**2**). Nevertheless, the study found that there was a significant correlation between BMI and MASLD grading, as most cases of grade II were overweight and obese classes (22/32) patients and all grade III MASLD patients were obese class II and III (3/3) (*p* = 0.004) as shown in Fig. (**3**).

The study found that the prevalence of fatty liver was higher in obese and overweight DM than in patients with normal BMI, as shown in Fig. (**4**).

## 4 DISCUSSION

The data suggest that MASLD tends to be more prevalent in patients with T2DM, maybe because of the common metabolic risk factors in both conditions [20, 21]. Additionally, it demonstrates a greater incidence of MASLD in the Saudi population (68.4%) compared to data from Chinese cohorts [22-24], where the prevalence of MASLD varies from 45.4% to 56.6%. Furthermore, compared to an Italian study that reported a 72.1% incidence of MASLD in T2DM patients, the prevalence in Saudi participants is slightly lower by 3.6%. This study's findings, while significant, are lower than a Romanian study that reported a higher prevalence of around 18.6% (68.4% in this study *versus* 87.1% in a Romanian study) [25]. These variances are essential for comprehending the worldwide distribution of MASLD and emphasize the significance of considering regional and racial differences when interpreting these findings.

Moreover, the data showed a significant difference in MASLD prevalence between uncontrolled and controlled diabetic patients. It was found that the probability of developing MASLD is three times higher in patients with uncontrolled diabetes, confirming a significant correlation between MASLD and individuals with uncontrolled diabetes, supported by a previous study [26]. These findings implicate that controlling diabetes effectively could reduce the risk of liver-related complications.

The study further indicates the increased prevalence of MASLD in males than females, with a difference of 3%. However, no significant difference was observed in the severity of MASLD between the genders. This aligns with prior research indicating a lack of significant gender-based differences in MASLD prevalence [25]. Hormonal and lipid metabolic profiles, including the triglyceride/high-density lipoprotein cholesterol ratio, are confirmed to vary between males and females. These differences could significantly influence the prevalence and progression of MASLD. However, these factors were not the primary focus of this study, indicating that further investigation into these gender-specific factors is essential to fully understand their impact on MASLD [22, 24, 27].

Obesity is a major risk factor in the development of T2DM, metabolic syndrome, and MASLD. A significant correlation was observed between the incidence of MASLD and BMI in patients diagnosed with T2DM. A large study conducted in Korea, including 29,994 people, found that 12.6% of non-obese participants had MASLD, whereas the prevalence was much higher at 50.1% among the obese group. Our findings highlight the strong link between MASLD and obesity, recognizing that lean individuals can develop hepatic steatosis as a prelude to increased BMI [28]. A study in South China also revealed a high prevalence of MASLD among overweight or obese individuals with T2DM [24].

Moreover, the work conducted by Ali *et al*. provides evidence for the predictive significance of BMI, specifically when it is above 24.5, in the diagnosis of MASLD in patients with T2DM. The study found a high accuracy rate of 96.8% [29]. This study found similar results, demonstrating that BMI is an essential determinant of MASLD in T2DM. These results indicate a significant association between obesity and the development of MASLD, especially in individuals with T2DM, emphasizing the importance of prioritizing obesity as a key focus for treatments.

MASLD has been linked to the continuous deterioration of metabolic abnormalities [30, 31]. A research indicates that the duration of diabetes plays a significant role in the development of MASLD, with a notable increase in MASLD prevalence in individuals with prolonged diabetes duration [32]. Contrarily, some studies suggest an inverse relationship between the duration of diabetes and MASLD, indicating that individuals with severe MASLD (grade 3) tend to be younger with a shorter history of diabetes [33, 34]. The current study observed that individuals with a diabetes history of no more than 10 years exhibited a 34% higher incidence of MASLD than those with a longer diabetes history. However, the difference was not statistically significant. This could be due to the random sampling method and the fact that a large proportion of participants (80 out of 115) presented with mild MASLD (grade I), with only three cases of severe MASLD (grade III).

A significant differences in liver size were observed across patients with different levels of T2DM treatment. Patients with uncontrolled DM demonstrated a greater liver size in comparison to those with controlled DM. The mean difference in liver size between the two groups was statistically significant, indicating a potential impact of uncontrolled DM on liver enlargement.

A limitation of this study is the small sample size and composition, with a predominance of mild fatty liver disease cases (grade 1) and minimal severe cases (grade 3). A follow-up study with a more stratified sample is recommended to better understand the progression of MASLD as a coexistence in T2DM Patients. Additionally, using a random sampling method may not have adequately captured the diversity and complexity of the diabetic population, potentially influencing the generalizability of the findings. However, this foundational work sets the stage for future research in the region, offering a crucial baseline for subsequent studies to build upon and expand our understanding of MASLD in the context of diabetes.

## CONCLUSION

In conclusion, our study revealed a significant correlation between uncontrolled diabetes and the prevalence of MASLD, with most patients exhibiting diffuse fatty liver. Interestingly, no notable association between the duration of diabetes and the severity of MASLD, nor between MASLD grading and gender, was observed. However, a higher BMI was correlated with increased MASLD grading.

Furthermore, patients with uncontrolled diabetes exhibited larger liver sizes, highlighting the potential impact of glycemic control on liver health. This underscores the critical importance of effective diabetes management in mitigating the risk of MASLD.

Therefore, to mitigate MASLD in T2DM patients, it is necessary to prioritize comprehensive health education for diet control and weight management, routine MASLD screening, particularly in those with abnormal BMI, and ensuring early detection and proper management to alleviate MASLD burden.

## Figures and Tables

**Fig. (1):**
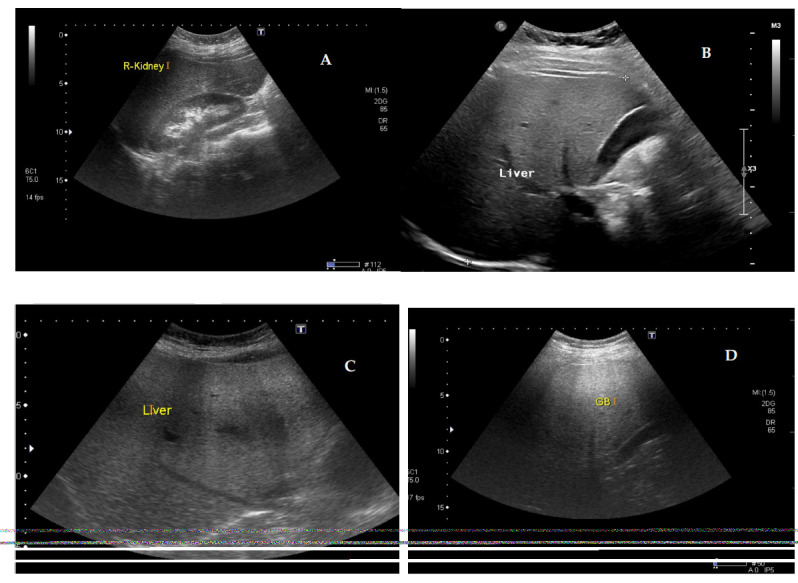
Classification of hepatic steatosis in B-mode ultrasound, ranging from normal (grade 0) to severe (grade 3), as illustrated in images **A** (G0), **B** (G1), **C** (G2), and **D** (G3).

**Fig. (2):**
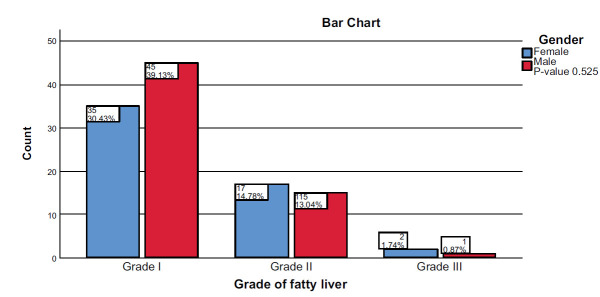
Correlation between MASLD grades and gender.

**Fig. (3):**
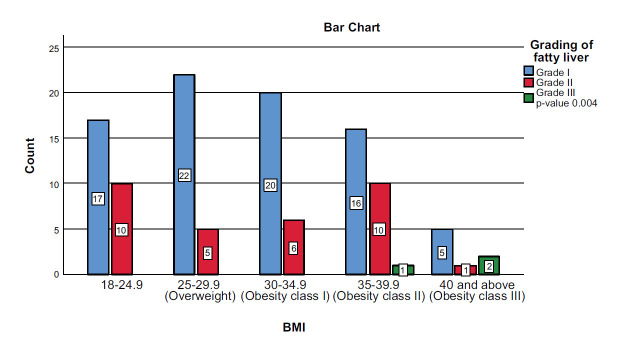
Correlation between MASLD grades and BMI.

**Fig. (4):**
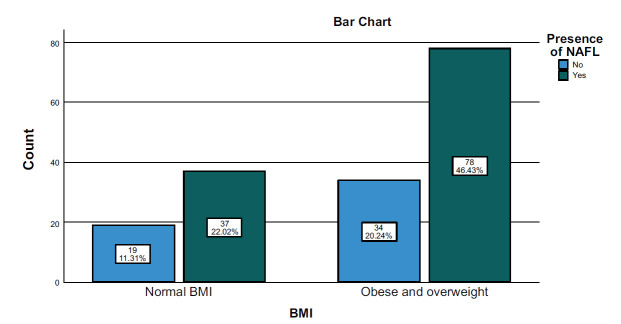
Correlation between the presence of MASLD and BMI.

**Table 1:** Distribution of the patients according to the sociodemographic and clinical characteristics.

**Variable**	**Characteristics**	**Frequency**	**Percentage**
**Age**	20-39	21	12.5
	40-59	73	43.5
	60-79	63	37.5
	80-99	11	6.5
**Gender**	Male	88	52.4
	Female	80	47.6
**BMI**	Normal	41	24.4
	Overweight	40	23.8
	Obesity class I	43	25.6
	Obesity class II	33	19.6
	Obesity class III	11	6.6
**Education**	Illiterate	7	4.2
	Intermediate	7	4.2
	Primary	104	61.9
	Secondary	29	17.3
	University	21	12.5
**Smoking history**	Current smoker	25	14.9
	Ex-Smoker	12	7.1
	Non-Smoker	131	78.0
**Treatment**	Both	72	42.9
	Insulin	15	8.9
	None	3	1.8
	Oral Hypoglycemic	78	46.4
**Duration**	Less than 1 year	32	19.0
	1-10 years	81	48.2
	11-20 years	38	22.6
	More than 20 years	17	10.1
**Diabetes status**	Control	26	15.5
	Uncontrolled	142	84.5
**Marital status**	Divorced	2	1.2
	Married	133	79.2
	Single	12	7.1
	Widow	21	12.5

**Table 2:** Distribution of the patients according to their ultrasound analysis.

**Variable**	**Characteristics**	**Frequency**	**Percentage**
**Fatty liver**	Yes	115	68.4
	No	53	31.6
**Type**	Diffuse	112	97.4
	Focal	3	2.6
**Grading**	I	80	69.6
	II	32	27.8
	III	3	2.6

**Table 3:** The correlation between MASLD and patients classified with controlled *versus* uncontrolled DM.

**Diabetes Status**	MASLD	**Normal**	**Total**	***X* ^2^**	**OR**	***p*-value**
**Uncontrolled**	103	39	142	0.913627	3.081, 95% CI (1.19-7.94)	0.015
**Controlled**	12	14	26			
**Total**	115	53	168			

**Table 4:** The correlation between MASLD grades and the duration of DM.

**Duration of DM**	**MASLD Grades**			**Total**	***p*-value**
	**Grade 1**	**Grade 2**	**Grade 3**		
**≤ 10 years**	55	19	3	77 (67%)	0.297
**> 10 years**	25	13	0	38 (33%)	
**Total**	80	32	3	115	

**Table 5:** Independent sample t-test to assess the liver size in uncontrolled and controlled DM patients.

**Status**	**Mean ± Std. Deviation**	**Std. Error Mean**	***p*-value, (95% CI)**
**Uncontrolled**	13.6±1.43	0.1204	0.032 (0.053-1.12)
**Controlled**	13.0±1.2	0.2353	

## Data Availability

The datasets used and analyzed during the current study are available from the corresponding author upon reasonable request.

## LIST OF ABBREVIATIONS

MASLD: Metabolic dysfunction-associated steatotic liver disease
NAFLD: Nonalcoholic fatty liver disease
EASL: European Association for the Study of the Liver
PDFF: Proton density fat fraction
DM: Diabetes Mellitus
OR: Odds ratio

## STANDARDS OF REPORTING

STROBE guidelines were followed.
